# Multiple Functions of Carbon Additives in NASICON-Type Electrodes for Stabilizing the Sodium Storage Performance

**DOI:** 10.3390/molecules30173547

**Published:** 2025-08-29

**Authors:** Trajche Tushev, Sonya Harizanova, Maria Shipochka, Radostina Stoyanova, Violeta Koleva

**Affiliations:** 1 Institute of General and Inorganic Chemistry, Bulgarian Academy of Sciences, 1113 Sofia, Bulgaria; tushev@svr.igic.bas.bg (T.T.); sonya@svr.igic.bas.bg (S.H.); shipochka@svr.igic.bas.bg (M.S.); radstoy@svr.igic.bas.bg (R.S.); 2 National Centre of Excellence Mechatronics and Clean Technologies, 1113 Sofia, Bulgaria

**Keywords:** sodium-ion batteries, mixed phosphate-sulphate electrodes, NaFeVPO_4_(SO_4_)_2_, rGO-based composite, carbon black-based composite, electrode stability, cycling stability, rate capability

## Abstract

Recently, there has been increased interest in NASICON-type electrodes for sodium-ion batteries due to their unique combination of intercalation properties, low cost, and safety. However, their commercialization is hindered by the low electrical conductivity. One strategy to overcome this issue is to integrate NASICON materials with carbon additives. This study shows that carbon additives improve the sodium storage performance of a NASICON-type electrode in various ways, depending on the additives’ functional groups, texture, and conductivity properties. The proof-of-concept is based on a multi-electron phospho-sulphate electrode, NaFeVPO_4_(SO_4_)_2_ (NFVPS) mixed with carbon black (C) and reduced graphene oxide (rGO). Carbon-coated samples are obtained via a simple ball milling procedure followed by thermal treatment in an argon flow. Sodium storage in the composites occurs through capacitive and Faradaic reactions. The Faradaic reaction is facilitated at the carbon black composite, while the capacitive reaction dominates for the rGO composite. NFVPS operates through two-electron reactions at 20 °C, while the increased temperatures favor the three-electron reaction. The rGO composite outperforms the carbon black composite in terms of cycling stability and rate capability at 20 and 40 °C. The role of the rGO and carbon black in electrochemical performance is discussed based on the different reactivity of hydroxyl/epoxide and carbonyl functional groups with the electrolyte salt, NaPF_6_, and the solvent, polypropylene carbonate.

## 1. Introduction

Sodium-ion batteries (NIBs) are emerging as competitive alternatives to lithium-ion systems, combining low cost and inherent safety with a comparable intercalation chemistry [1]. In this context, NASICON-derived materials with compositions A_x_M_y_(XO_4_)_n_ (A  =  Na, Li, K, M  =  Fe, Mn, Co, etc. and X  =  P, S, Si, Mo, etc.) stand out as especially attractive electrode candidates [2]. The NASICON structure allows for the rapid diffusion of the Na^+^ ions, enabling fast and reversible sodium intercalation without significant structural degradation [3]. Although NASICONs deliver excellent cycling stability and high rate capability, their intrinsically low electronic conductivity demands tailored electrode architectures to unlock optimal sodium-storage performance [4,5,6]. Three principal strategies address these limitations: structural engineering, morphology design and surface modification [2,4,5,6]. Structural engineering tunes the electronic structure via selective single-ion or multi-ion doping [7,8,9], while morphology design manipulates the nucleation and growth of NASICON particles to expose the crystallographic planes or axes where sodium intercalation occurs preferentially [10,11]. In comparison with these two strategies, surface modification enhances the overall conductivity by integrating conductive, porous carbon additives onto the phosphate framework [12,13,14,15]. Surface modification is more effective because it affects electronic conductivity, electrode-electrolyte interactions, electrolyte wetting of the electrode, corrosion resistance, thermal stability, and leaching of transition metal ions from NASICON phase.

Due to variety in the texture and conductivity properties, low cost, and easy synthesis procedure, the use of carbonaceous materials for the surface modification of NASICON-type electrodes has become the main research topic [4,5,6]. Through hydrothermal assisted sol-gel methods, electrospinning technique and high-boiling organic solvent-assisted colloidal synthesis, the core-shell nanocomposite, Na_3_V_2_(PO_4_)_3_@C, having excellent cycling stability, has been synthesized [16,17,18,19,20]. Mechanical ball milling followed by thermal reduction at various temperatures has also proven effective in preparing Na_3_V_2_(PO_4_)_3_@C with enhanced performance [21]. Another part of the study involves carbon doping with nitrogen and/or boron to accelerate the Na^+^ transport in the carbon layer, thereby improving the rate capability and cycling stability of NASICON-type electrodes [22]. Pyrolysis of organic precursors is a common method for producing amorphous carbon coating [23]. This coating is then treated (namely by chemical vapour deposition, etc.) to form highly conductive, graphene-like coated carbon (for example, carbon-coated Na_3_V_2_(PO_4_)_3_ with 0.5% amorphous embedded in rGO with about 3%) [24]. The integration of the NASICON-material into flexible carbon network (such as carbon cloth, carbon nanofibers/nanotubes, graphene paper, etc.) has also been reported as effective method for improving the total conductivity of the electrode [20,25,26,27,28]. In summary, much research has been focused on the electrochemical performance of carbon-modified NASICON-type electrodes, while little research has addressed their total thermal stability [29]. Despite the good thermal stability of NASICON-type materials [30] the carbon additives in modified NASICON electrodes usually have lower thermal stability, resulting in lower total thermal stability. This is an important issue for the practical application of NASICON-type electrodes.

The present study is focused on the tracking the effect of carbon additive on both the thermal stability and sodium storage performance of multi-electron NASICON-type electrodes. The multi-electron phospho-sulphate, NaFeVPO_4_(SO_4_)_2_, is selected as the NASICON-type electrode because it is able to reversibly intercalate from two to three moles of Na^+^ ions between 1.5 and 4.2 volts [31,32]. This process is accompanied by limited variation in the lattice volume (up to 5.2%) [31]. In general, the sulphate salts are less thermally stable than the phosphate salts, but from electrochemical point of view the presence of SO_4_^2−^ groups in the electrode material is valuable since SO_4_^2−^ group increases the potential of the redox reactions, thus increasing the power density of the cells [31,32]. The carbon additives comprise the carbon black and reduced graphene oxide (rGO). These additives have been shown to be effective for improving the electrochemical performance of iron phospho-sulphate, NaFe_2_PO_4_(SO_4_)_2_@rGO, and mixed iron-vanadium and phospho-sulphate, NaFeVPO_4_(SO_4_)_2_ [31,33]. When CNT is used, the composite NaFe_1.6_V_0.4_(PO_4_)(SO_4_)_2_@CNT operates at a working voltage of approximately 3 V due to the Fe^3+^/Fe^2+^ couple [34], which is contrary to the previous findings [31,32,33]. The carbon coated NaFeVPO_4_(SO_4_)_2_ samples are obtained via a simple ball milling procedure followed by thermal treatment at 400 °C in an argon flow. The storage performance was analysed in model sodium half-cells at elevated temperatures. Ex-situ XRD and XPS analyses allow us to distinguish the roles of carbon black and rGO in the electrochemical performance of NaFeVPO_4_(SO_4_)_2_ (hereinafter referred to as NFVPS).

## 2. Results

### 2.1. Characterization of NFVPS/rGO and NFVPS/C Composites

The active phase NaFeVPO_4_(SO_4_)_2_ (NFVPS) adopts NASICON-type structure in trigonal space group *R*-3 (Figure S1a) where corner shared (Fe,V)O_6_ octahedra and (P,S)O_4_ tetrahedra form three-dimensional channels for the sodium ions movements [31]. The lattice parameters are *a* = 8.4691 (1) Å and *c* = 22.0162 (1) Å (Table S1). After ball milling of NFVPS with 15 wt.% carbon additives, carbon black and rGO (denoted as NFVPS/C and NFVPS/rGO, respectively), the NASICON crystal structure remains intact, and the lattice parameters approach those of untreated NFVPS (see Figure S1 and Table S1). The crystallinity of the carbon-modified samples is also preserved. The crystallite size varies from 53 to 48 nm.

**Figure 1:**
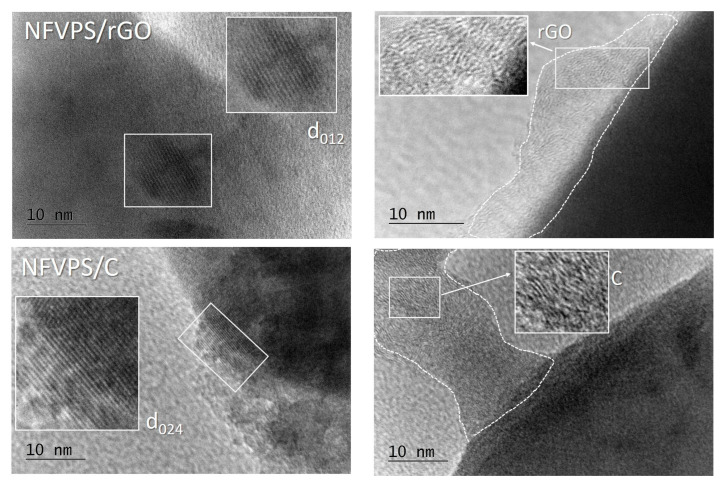
HR-TEM images of NFVPS/rGO (**top**) and NFVPS/C (**bottom**).

The presence of carbon additives in the composites is evident from the HR-TEM images (Figure 1).

The carbon additives cover the phosphate-sulphate particles, but the carbon layers are inhomogeneous with thickness between 5 and 10 nm for NFVPS/rGO, and between 5 and 25 nm, for NFVPS/C. For the latter composite, the boundary between the NFVPS phase and carbon black cannot be clearly distinguished. It is of importance that both rGO and carbon black are in good interphase contact with the phospho-sulphate phase. Additionally, HR-TEM image of NFVPS/rGO reveals distinct fringes, with a calculated interplanar spacing of 0.60 nm that corresponds to the (012) plane of the trigonal NFVPS phase. This indicates that both the crystal structure and crystallinity of the phosphate-sulphate phase in the composite with rGO are intact. The same applies to the NFVPS/C composite, where the HR-TEM image shows lattice fringes with spacing of 0.30 nm corresponding to the (024) plane of the NFVPS lattice (Figure 1).

**Figure 2:**
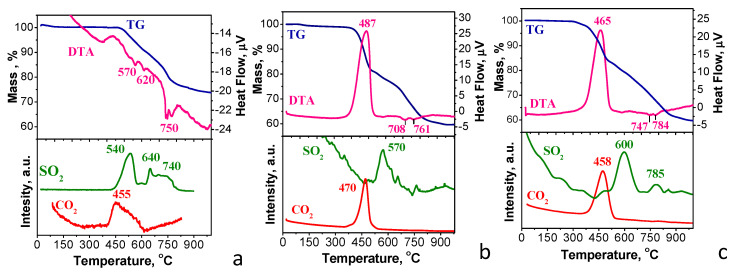
TG/DTA/MS curves of NFVPS (**a**), NFVPS/C (**b**) and NFVPS/rGO (**c**).

To determine the thermal stability of the carbon-modified NFVPS samples, the simultaneous thermogravimetry/differential thermal analysis/mass spectrometry analyses (TG/DTA/MS) were undertaken up to 1000 °C (Figure 2). The thermal decomposition of NFVPS begins at 440 °C with release of SO_2_ gas and this process is accompanied by multiple endothermic effects on the DTA curve extending up to 830 °C (Figure 2a). The registration of CO_2_ gas evolved between 430 and 650 °C reveals that the NFVPS sample contains residual in-situ-carbon generated from the citric acid presented in the precursor composition. The total mass loss for NFVPS is 26.5 mass %. These data reveal that the decomposition of NFVPS is a multistage process involving at least three stages, as determined by thermogravimetric analysis. Between 450 and 600 °C, NFVPS is probably decayed to Fe_2_(SO_4_)_3_ and Na_3_V_2_(PO_4_)_3_, releasing SO_2_ (mass loss of 9 mass % versus experimental value of 10%). Then, the in situ formed Fe_2_(SO_4_)_3_ decomposes to iron oxide polymorphs, releasing SO_2_, culminating in α-Fe_2_O_3_ at 700 °C [35]. Within this temperature range, V^3+^ ions can also be oxidized to V^4+^ ions [36], thus contributing to the decomposition of in-situ formed Na_3_V_2_(PO_4_)_3_. Above 700 °C, the final stage of decomposition takes place.

In comparison with carbon-free NFVPS sample, the decomposition of carbon-modified samples NFVPS/C and NFVPS/rGO starts at lower temperature (i.e., about 380 °C). This is related with the lower thermal stability of the carbon additives (Figure 2b,c). Moreover, the TG curves for NFVPS/C and NFVPS/rGO show two-step mass loss processes. These processes are accompanied by the initial release of CO_2_ up to 500 °C, followed by the release of SO_2_ up to 830 °C. The total mass loss reaches 40 mass %. Notably, SO_2_ is released at a higher temperature in the composites than in the carbon-free NFVPS: 570 °C and 600 °C for NFVPS/C and NFVPS/rGO vs. 540 °C for NFVPS. This indicates that the presence of carbon additives on the NFVPS surface impacts the initial stage of NFVPS decomposition. This can be explained by the ability of carbonaceous materials to absorb SO_2_ [37]. This retards the release of SO_2_ during the NFVPS decomposition. It is worth mentioning that SO_2_ is a toxic gas. Therefore, the enhanced temperature of SO_2_ release in the composite supports its possible practical application. Once the carbon additive burns completely, the decomposition of the NFVPS becomes uncontrolled. Another difference in the thermal behavior of the carbon-free and carbon-modified NFVPS can be seen in the DTA curves (Figure 2). Due to the small amount of in-situ generated carbon in NFVPS, the endothermic process of the NFVPS decomposition dominates over the carbon oxidation and the overall thermal reaction registered on DTA curve is endothermic one (Figure 2a). The opposite is observed for the two composites. Due to the large amount of the carbon additive in the composites (15%) the exothermic process of the carbon oxidation to CO_2_ dominates over the endothermic sulphate decomposition to SO_2_ (Figure 2b,c).

**Figure 3:**
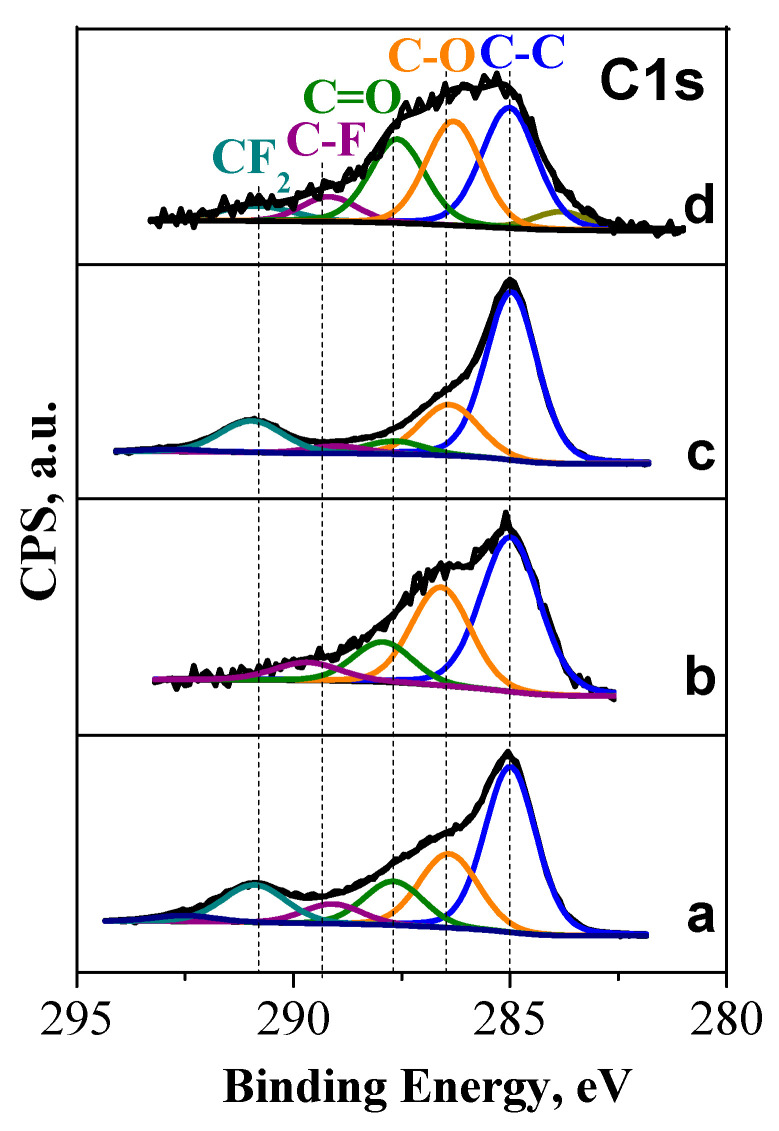
XPS spectra in the region of C1s binding energies of pristine NFVPS/rGO electrode (**a**) and after 200 cycles in Na-half cell (100 cycles at 20 °C and subsequent 100 cycles at 40 °C with a rate C/2) (**b**); pristine NFVPS/C electrode (**c**) and after 200 cycles in Na-half cell (100 cycles at 20 °C and subsequent 100 cycles at 40 °C with a rate C/2) (**d**).

The difference in the thermal properties of the composites can be related with the functional groups stabilized in the carbon additives. Figure 3 shows the XPS spectra of NFVPS/C and NFVPS/rGO in the region of the C1s binding energy.

The C1s spectra of NFVPS/C and NFVPS/rGO consist of a broad, asymmetric band that can be deconvoluted into peaks centered at 285.0, 286.5, 287.6, 289.3, and 290.8 eV. These peaks can be assigned to C–C bonds in the carbon network as well as to single- and double-bonded carbon-oxygen bonds: 286.5 eV for C–O and 287.6 eV for C=O. (It is worth mentioning that the peaks appearing above 289 eV are due to the C–F and CF_2_ bonds in the PVDF used as a binder). The relative amounts of the hydroxyl/epoxide and carbonyl groups are listed on Table 1. The element contents determined from XPS spectra are given in Table S2.

The comparison evidences that the epoxy and hydroxyl groups dominate the composite surface with carbon black. Meanwhile, both hydroxyl/epoxide groups and carbonyl groups contribute to the functionality of the composite with rGO. In comparison with C1s spectra, the assignments of O1s peaks to O species in the literature are not the straightforward procedure as in the case of C1s spectra [38,39]. However, the main conclusions from C1s spectra are supported by O1s spectra (Figure S2): the bands at around 532 and 533.0 eV that can be assigned to double-bonded O atoms in esters, carbonates, and acids, as well as to single-bonded O atoms in ketones, ethers, and alcohols [38,39]. Because the sample NFVPS/rGO has a higher concentration of the oxygen functional groups, it undergoes pyrolysis at a slightly lower temperature than that of NFVPS/C: 458 °C vs. 470 °C, respectively (Figure 2).

**Figure 4:**
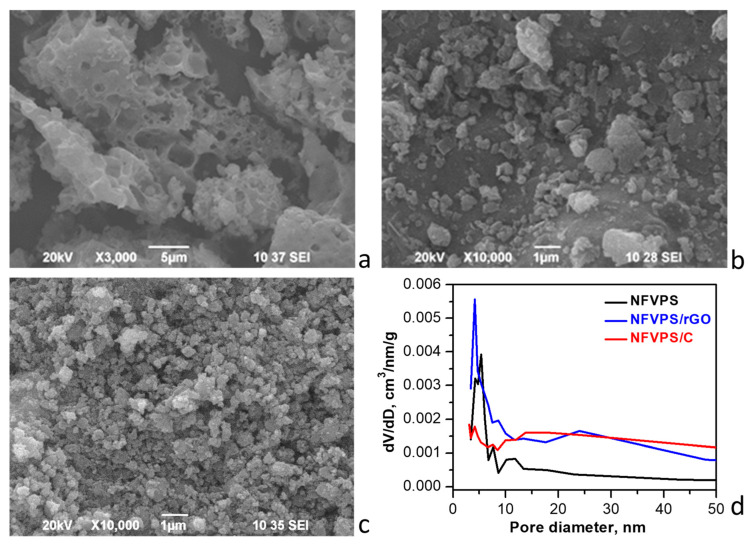
SEM images of NFVPS (**a**), NFVPS/rGO (**b**) and NFVPS/C (**c**); Pore size distribution curves (**d**).

Figure 4 and Figure S3 compare both the morphologies and porous characteristics of NFVPS and the carbon composites.

For pristine NFVPS the SEM image shows large micrometers aggregates (above 5–10 μm) having big holes on their surface, the latter being formed from the release of gases during the thermal decomposition of the organic precursor (Figure 4). After ball milling of NFVPS with carbon additives, the aggregates break apart and become smaller. The sizes of the aggregates vary widely when rGO is added, ranging from 0.2 to 2–3 µm. In contrast, carbon black yields more regular aggregates with a close size distribution around 0.5 µm. This indicates that carbon additives stick to the NFVPS during ball milling (Figure 4). Furthermore, the nitrogen adsorption isotherms reveal a significant difference in the porosity of the three samples resulting from the presence of the carbon additives (Figure S3). The individual rGO and carbon black materials have very different texture properties as previously reported [40] and this reflects on the composites porosity. Because of high specific surface areas of the carbon additives (363 cm^2^/g for rGO and 49 cm^2^/g for carbon black [40]), the composites exhibit a two- to threefold increase in specific surface area compared to NFVPS: 31 cm^2^/g for NFVPS/rGO and 17 cm^2^/g for NFVPS/C versus 9 cm^2^/g for NFVPS [31]. In the same order, the total pore volumes substantially increase: 0.12 cm^3^/g for NFVPS/rGO and 0.10 cm^3^/g for NFVPS/C vs. 0.03 cm^3^/g for the uncoated sample.

Regarding pore size distribution, the three samples exhibit a large part of the mesopores with diameters in the range of 3–9 nm, as for NFVPS these pores are the predominant ones (Figure 4d). In contrast, for the composites there is additional contribution of larger mesopores: with diameters between 9 and 50 nm for NFVPS/C and between 15 and 50 nm for NFVPS/rGO, which are associated with the carbon additives [40]. From nitrogen adsorption studies it can be concluded that the high porosity of the composites could facilitate the electrolyte penetration in the electrode materials, thus enhancing the kinetics of sodium ion diffusion during the electrochemical cycling. On the other hand, the pore structure of NFVPS/rGO is more developed than that of NFVPS/C which is a prerequisite for its better electrochemical performance.

### 2.2. Electrochemical Studies

**Figure 5:**
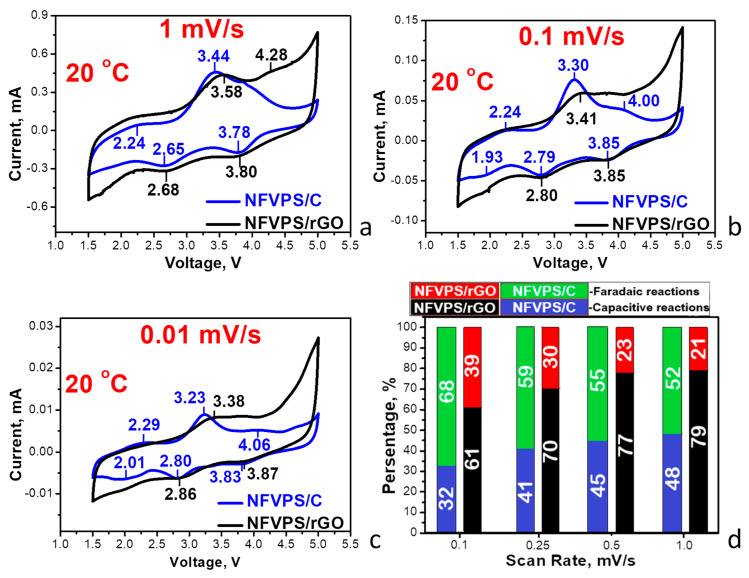
CV curves with scanning rates of 1 mV/s (**a**), 0.1 mV/s (**b**) and 0.01 mV/s (**c**) between 1.5 and 5.0 V for NFVPS/C and NFVPS/rGO in sodium-half cells at 20 °C. Contribution of Faradaic and Capacitive reactions (**d**).

Figure 5 and Figure S4 compare the CV curves of NFVPS/rGO and NFVPS/C composites at different scanning rate.

Between 1.5 and 5.0 V, the CV curves display a series of redox peaks, corresponding to the V^3+^/V^2+/4+^ and Fe^3+^/Fe^2+^ couples [31]. From the one hand, this evidences that electrochemical reaction proceeds in the same way for the composites. From the other hand, the redox peaks are more pronounced in the NFVPS/C composite, thus implying that capacitive reactions mainly occurs for the composite with rGO. Taking into account the correlation between the current and scanning rate [41], the contribution of the Faradaic and capacitive reactions towards sodium storage is evaluated (Figure 5d). The comparison shows that the Faradaic reaction dominates the electrochemical reaction at scanning rates lower than 1 mV/s, while the capacitive reaction becomes dominant at rates above 1 mV/s. Among the composites, the Faradaic reaction is facilitated by the composite with carbon black, while capacitive reaction dominates for the composite with rGO [42,43,44]. This can also be related with well-developed pore structure of NFVPS/rGO.

**Figure 6:**
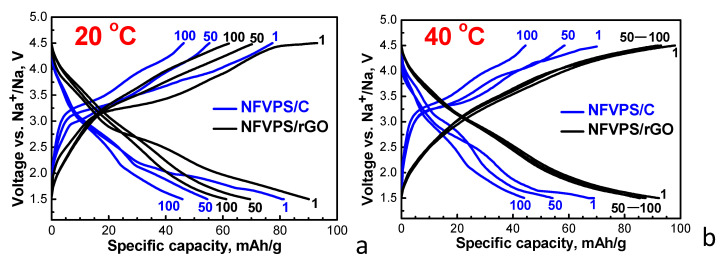
Charge-discharge curves (1st, 50th and 100th cycle) with a C/2 rate between 1.5 and 4.5 V of NFVPS/rGO and NFVPS/C at 20 °C (**a**) and 40 °C (**b**). The electrolyte is NaPF_6_ in propylene carbonate (PC).

To compare the sodium storage performance of NFVPS/rGO and NFVPS/C, a cycling stability test was carried out at 20 °C and 40 °C at a C/2 rate. The testing protocol comprises the first 100 cycles at 20 °C and the next 100 cycles at 40 °C. The corresponding charge/discharge curves at a given cycle are presented on Figure 6.

The charge/discharge curves of the two composites display similar profiles between 1.5 and 4.5 V, once again demonstrating that the sodium storage mechanism is the same as that observed in CV experiments (Figure 5).

**Figure 7:**
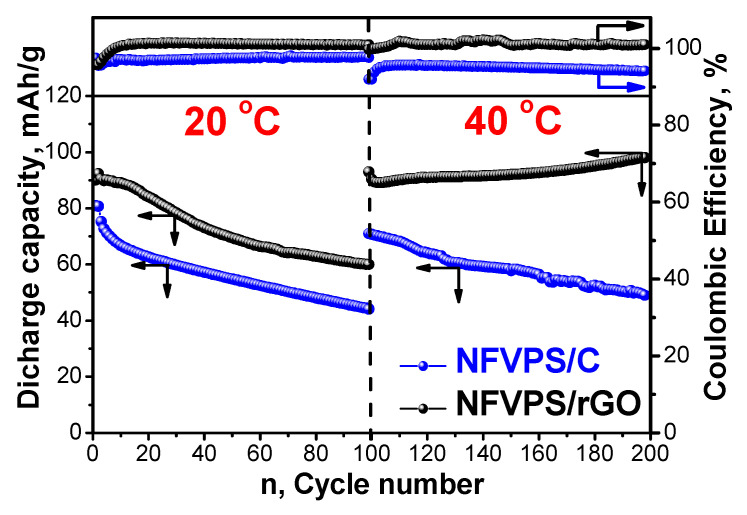
Cycling stability and Coulombic efficiency of NFVPS/rGO and NFVPS/C in Na half-cells at 20 °C and 40 °C at a C/2 rate. The cells operate firstly at 20 °C (cycles from 1 to 100) and then at 40 °C (cycles from 101 to 200).

At 20 °C, the first discharge capacity of both NFVPS/C and NFVPS/rGO is comparable: 80 mAh/g and 90 mAh/g, respectively (Figure 6). The discharge capacity of NFVPS/C decreases monotonously upon cycling, while the capacity of NFVPS/rGO quickly decreases up to 50 cycles and remains almost the same between 50 and 100 cycles (Figure 7). The cycling stability of NFVPS/C and NFVPS/rGO are around 55 and 70%, respectively (Figure 7). The Coulombic efficiency of NFVPS/C approaches 100%, whereas the efficiency of NFVPS/rGO slightly deviates from 100% (Figure 7).

After increasing the temperature of the cells from 20 to 40 °C, the discharge capacity for both NFVPS/C and NFVPS/rGO increases reaching a magnitude of 70 and 90 mAh/g.

As in the case of the 20 °C experiment, the discharge capacity of NFVPS/C continues to decrease upon cycling, while the discharge capacity of NFVPS/rGO slightly increases from 90 to 98 mAh/g after 100 cycles. This is related with an already formed surface layer at 20 °C, which appears to prevent the further interaction of the electrode with the electrolyte at 40 °C [31]. It is important to note that the cycling stability of both samples is better at 40 °C than at 20 °C: 70% for NFVPS/C and 95% for NFVPS/rGO, respectively. Once again, the Coulombic efficiency is better for NFVPS/C. Thus, the electrochemical protocol discloses a significant difference in storage performance between NFVPS/C and NFVPS/rGO composites.

**Figure 8:**
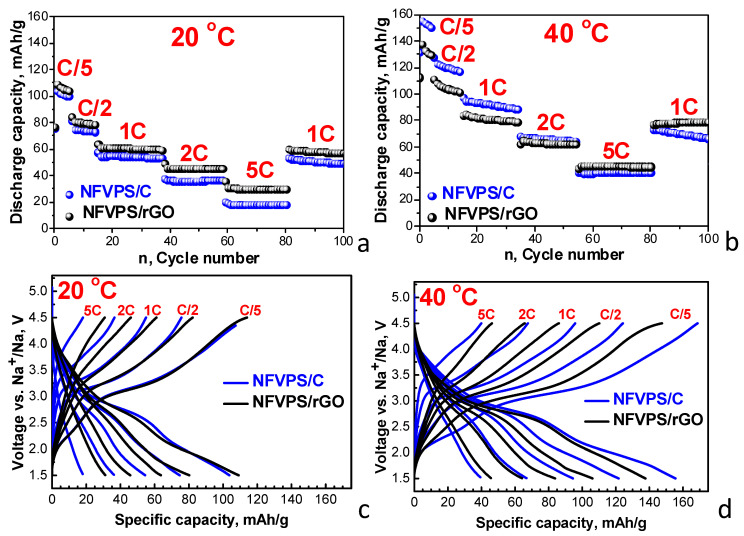
Rate capability in Na half-cells of NFVPS/rGO and NFVPS/C at rates ranging from C/10 to 5C and then to 1C at 20 °C (**a**) and 40 °C (**b**). Coulombic efficiencies are also shown (**a**,**b**); Second charge-discharge curves of NFVPS/rGO and NFVPS/C recorded at rates from C/10 to 5C at 20 °C (**c**) and 40 °C (**d**).

The next electrochemical protocol is designed to determine the rate capability at elevated temperatures (Figure 8).

At 20 °C, NFVPS/C and NFVPS/rGO deliver almost the same discharge capacity at the slowest rate (i.e., varying between 110 and 100 mAh/g at a C/5 rate). This provides evidence for a two-electron reaction involving Fe^3+^/Fe^2+^ and V^3+^/V^4+^ couples occurring at NFVPS, which has a theoretical capacity of 128 mAh/g. At the fastest rate, NFVPS/rGO outperforms NFVPS/C: the discharge capacity is of 30 mAh/g versus 15 mAh/g at a 5C rate. When the rate returns from 5C to 1C, the capacity of NFVPS/rGO is restored to approximately 60 mAh/g and this capacity remains nearly unchanged after next 20 cycles. For NFVPS/C, capacity is nearly restored but decreases continuously with cycling. Furthermore, the capacity increases as temperature rises from 20 to 40 °C. At a C/5 rate, the initial capacities reach to 160 and 140 mAh/g for NFVPS/C and NFVPS/rGO, respectively. This suggests that the increased temperature favours the occurrence of a three-electron reaction involving Fe^3+^/Fe^2+^ and V^3+^/V^4+^/V^5+^ couples in NFVPS, which implies kinetic limitations for the three-electron reaction compared to the two-electron one. By increasing the rate from C/5 to 5C, the capacities of NFVPS/C and NFVPS/rGO become similar, around 40 mAh/g at 5C. This is another indication that the rate capability of NFVPS/rGO at 40 °C is better than that of NFVPS/C. Additionally, when the charging rate is returned from 5C to C/1, the capacities of NFVPS/rGO and NFVPS/C become close (77 mAh/g for NFVPS/rGO and 73 mAh/g for NFVPS/C). However, the capacity stability of NFVPS/rGO is much better than that of NFVPS/C after 20 cycles: 100% versus 92%, respectively. Therefore, regardless of the testing protocol, NFVPS/rGO outperforms NFVPS/C in terms of cycling stability and rate capability.

### 2.3. Ex-Situ Studies

**Figure 9:**
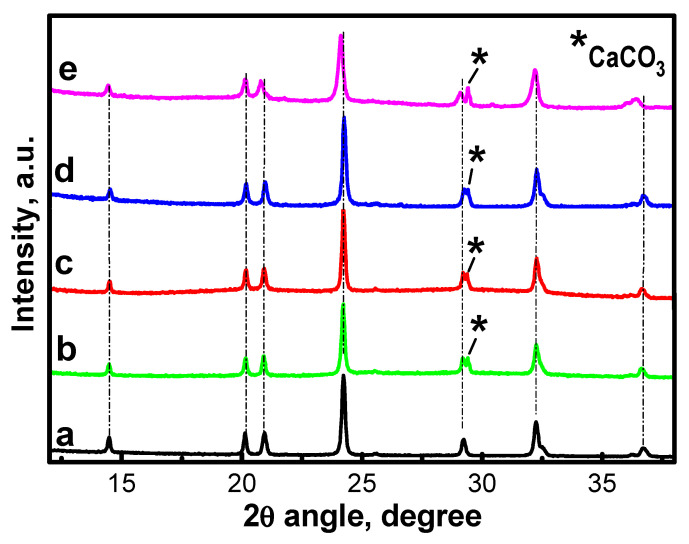
XRD pattern of powder NFVPS (**a**); Ex-situ XRD patterns of pristine NFVPS/rGO electrode (**b**) and after 200 cycles in Na-half cell (100 cycles at 20 °C and subsequent 100 cycles at 40 °C with a rate C/2) (**c**); pristine NFVPS/C electrode (**d**) and after 200 cycles in Na-half cell (100 cycles at 20 °C and subsequent 100 cycles at 40 °C with a rate C/2) (**e**). * CaCO_3_ from plastic holder.

The electrochemical performance of carbon-containing composites is due to the structural stability of NFVPS phase during the prolonged sodium extraction/insertion (Figure 9). For ex-situ XRD experiments, we analyzed the electrodes after 100 cycles at 20 °C, followed by 100 cycles at 40 °C, and then switch them off at 1.5 V (i.e., in the discharged state).

The ex-situ XRD patterns show that the NASICON-type structure of NFVPS is preserved with lattice parameters close to that for the pristine electrodes (Table S1). The structure stability of NFVPS is its important advantage from practical point of view. Apart from the structural stability, the electrode roughness also seems unchanged after prolonged cycling even at 40 °C as seen from the optical images (Figure S5).

The same electrodes are subjected to XPS analyses (Figure 9). This technique allows us to probe the surface of the cycled electrodes.

For the pristine electrodes, the spectra in the binding energy range of V2p_3/2_ and Fe2p consist of peaks centered at around 517 eV and 713 eV, corresponding to V^3+^ and Fe^3+^ ions. Additionally, the S2p and P2p spectra show peaks due to SO_4_ and PO_4_ groups. Within the Na1s binding energy range, the spectra are dominated by a single peak corresponding to sodium atoms bound to PO_4_ groups. After electrode cycling, the peaks due Na^+^ in PO_4_ environment, SO_4_ and PO_4_ groups retain their positions. This provides evidence for the stability of the SO_4_ and PO_4_ groups during the cycling. In the pristine electrodes Fe^3+^ ions are visible, but after cycling, both Fe^3+^ and Fe^2+^ present on the electrodes surfaces (discharged states). The spectra are barely visible in the V2p_3/2_ range, suggesting that thicker surface layers are formed on the cycled electrodes.

**Figure 10:**
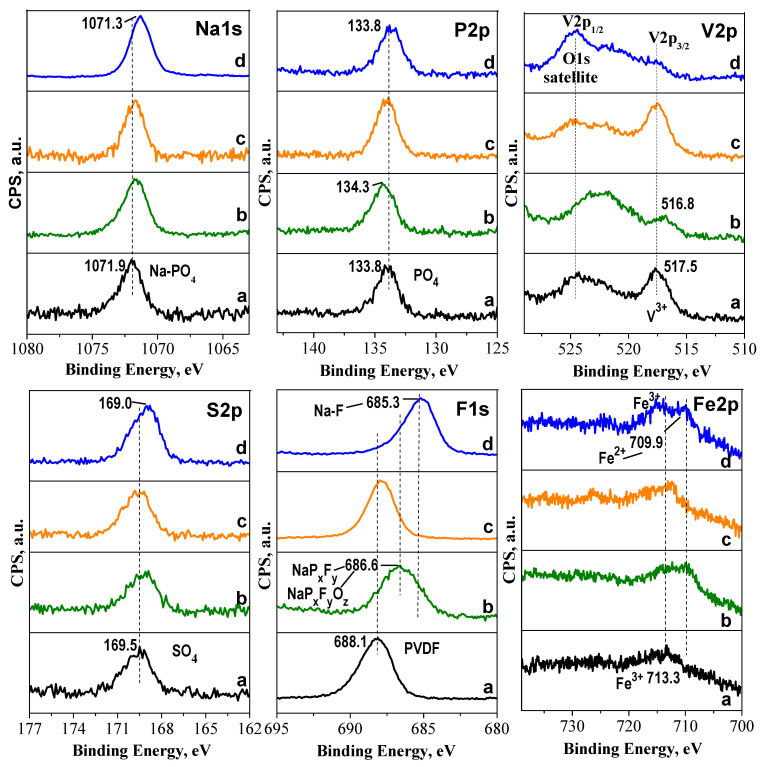
XPS spectra of pristine NFVPS/rGO electrode (**a**) and after 200 cycles in Na-half cell (100 cycles at 20 °C and subsequent 100 cycles at 40 °C with a rate C/2) (**b**); pristine NFVPS/C electrode (**c**) and after 200 cycles in Na-half cell (100 cycles at 20 °C and subsequent 100 cycles at 40 °C with a rate C/2) (**d**).

To analyze the electrochemically formed surface layers, the XPS spectra in the range of F1s and C1s are of interest (Figure 10). The F1s spectra of the pristine electrodes exhibit a band at 688.1 eV, which is typical for F atoms in PVDF (polyvinylidene fluoride) binder [45]. After cycling, this band shows significant downshift to 686.6 eV for NFVPS/rGO and to 685.3 eV for NFVPS/C (Figure 9). The lowest energy band at 685.3 eV observed for NFVPS/C can be assigned to the formation of fluorides (i.e., F bonded primarily to Na and NaP_x_F_y_) and/or oxidized fluoride species, P_x_F_y_O_z_ [46]. For NFVPS/rGO, the band position at 686.6 eV suggests for the deposition of oxidized fluoride species, P_x_F_y_O_z_, rather than fluorides [47,48]. The fluorine products detected on the electrode surface can be related with the reactivity of the electrolyte salt NaPF_6_ towards the carbon additives: the interaction of NaPF_6_ with carbon black in NFVPS/C yields mainly fluorides, while rGO in NFVPS/rGO facilitates the deposition of oxofluorides.

The C1s spectra of the electrodes are also changed after the electrode cycling (Figure 3, Table 1). This finding suggests that the electrolyte solvent propylene carbonate (PC) reacts with the electrode surface. Furthermore, the extent of the interaction with PC depends on the origin of the carbon additives. As shown in Table 1, the electrode surface of the composite with carbon black is enriched with hydroxyl/epoxide functional groups. In the presence of rGO, the electrode surface contains both hydroxyl/epoxide and carbonyl functional groups, with a slight prevalence of hydroxyl/epoxide groups. This can be interpreted in terms of the reactivity of the electrolyte solvent, PC. There are two options for the decomposition of PC [49]. The energetically favorable pathway is through ring opening of the PC molecule and formation of alkylcarbonates, which further degrade to carbonates and alkenes. The second option involves ring opening, which yields alkoxyalkyl compounds. This is followed by the formation of epoxides and CO_2_. According to the XPS study, it appears that PC degradation via the formation of alkoxyalkyl compounds proceeds more easily at the carbon black composite, while both PC degradation pathways occur on the rGO composite surface. The different reactivity of PC towards composite surfaces determines the composition of electrochemical surface layers, which in turn contribute to the cycling stability and rate capability. This is the first experimental observation for the effect of carbon additives on the degradation pathways of PC, which requires further experimental and theoretical studies.

## 3. Materials and Methods

### 3.1. Synthesis

The powder NaFeVPO_4_(SO_4_)_2_ (NFVPS) has been synthesized by a precursor method, following by annealing at 400 °C in Ar flow. The details are reported elsewhere [31]. Two types of carbon composites have been prepared, the one with traditional carbon black (Super C65, TIMCAL Ltd., Bodio, Switzerland) and another with rGO (Graphit Kropfmühl GmbH, Hauzenberg, Germany). A simple mechanical grinding method has been applied to obtain composites between NFVPS and 15 wt.% carbon additives, which are labelled as NFVPS/C and NFVPS/rGO. The ball-milling of the materials has been carried out for 4 h at a speed of 300 rpm using agate balls with ϕ of 10 mm, balls-to-powder ratio was 10:1. The grinding procedure has been performed by means of planetary mill “Pulverisette 6” (Fritsch GmbH, Idar-Oberstein, Germany). After the milling the composites have been annealed at 400 °C for 3 h under Ar flow.

### 3.2. Characterization Methods

Powder X-ray diffraction technique (XRD) has been used to check the structure, phase composition and purity of pristine NFVPS, composites and electrodes after the electrochemical cycling (Bruker Advance D8 diffractometer with CuKα radiation, Karlsruhe, Germany). The lattice parameters have been calculated by WinPLOTR program. The thermal stability of NFVPS and composites has been studied by simultaneous thermogravimetry and differential thermal analysis combined with mass spectrometry for gas analysis (TG/DTA/MS) at a heating rate of 10 °C/min under Ar atmosphere (LABSYSTM Evo apparatus, SETARAM, Caluireet-Cuire, France). The specific surface area and total pores volume of the samples have been calculated by BET method based on the nitrogen adsorption-desorption isotherms (77.4 K) obtained by NOVA 1200e device (Quantachrome, Boynton Beach, FL, USA). The morphology of the powder samples and electrodes has been observed by scanning electron microscopy (SEM) using JSM 6390 microscope (JEOL, Tokyo, Japan). HR-TEM analysis has been performed with a JEOL 2100 microscope (Tokyo, Japan) with a GATAN Orius 832 SC1000 camera (Plesantan, CA, USA). The DigitalMicrograph software version 2 (Gatan, Inc., Pleasanton, CA, USA) to calculate the HR-TEM images was used. To probe the chemical and electronic state of the elements on the electrode surfaces X-ray photoelectron spectroscopy (XPS) analysis was carried out (VG Escalab II system with Al K_α_ radiation, Thermo Fisher Scientific, Waltham, MA, USA). The C1s line of adventitious carbon at 285.0 eV was used as an internal standard to calibrate the binding energies. The photoelectron spectra were corrected by subtracting a Shirley-type background and they were quantified using the peak area and Scofield’s photo-ionization cross-section. The accuracy of the binding energy measured was ±0.1 eV. The optical images of the electrodes have been collected using ZEISS Stemi 508 stereo microscope (Carl Zeiss, Jena, Germany).

The electrochemical characterization has been performed in Swagelok type of sodium half-cells by means of 32 channel Biologic VMP-3e battery cycler (BioLogic, Seyssinet-Pariset, France). The potentiostatic and galvanostatic cycling tests have been carried out at 20 and 40 °C (KB-53 incubator, Binder GmbH, Tuttlingen, Germany).

The positive electrode contains 80% composite (NFVPS/C or NFVPS/rGO), 10% Super C65 carbon and 10% polyvinylidene fluoride (PVDF) which have been homogenized in N-methyl-2-pyrrolidone using planetary centrifugal mixer ARE-250 CE (THINKY, Tokyo, Japan). Then the slurry has been cast onto the carbon-coated aluminium folio (Goodfellow, Cambridge Ltd., Cambridge, UK) with a doctor blade film coater (ZAA 2600.A, Proceq SA, Schwerzenbach, Switzerland) and vacuum dried at 80 °C overnight. The electrode film with a thickness of 200 μm is then cut into 10 mm discs, pressed and vacuum dried at 120 °C for 10 h. The electrode loading is around 3 mg/cm^2^. The negative electrode consists of sodium metallic disc. The electrolyte solution is 1 M NaPF_6_ in PC (propylene carbonate) soaked in a glass fibre separator GF/D Whatman (Whatman International Ltd., Maidstone, UK). The sodium test cells have been assembled in an argon-filled glove box MB-Unilab Pro SP (1500/780) under controlled traces of O_2_ and H_2_O below 0.1 ppm (MBraun, Garching, Germany). The cyclic voltammograms (CV) have been scanned over the range 1.5–5.0 V (vs. Na^+^/Na) with different rates between 10 and 0.01 mV/s. The contributions of the capacitive and Faradaic reactions are determined using the relationship between total current (i in A) at a specific potential (V) and scanning rate (v in V/s) according to Equation (1) [41,50]:

i(V) = k_1_v + k_2_v^1/2^ (1)

where, k_1_ and k_2_ are adjustable parameters. In Equation (1), k_1_v and k_2_v^1/2^ correspond to the current attributed to capacitive reaction and diffusion-controlled reaction, respectively [41,50].

The galvanostatic tests in a voltage window of 1.5–4.5 V (vs. Na^+^/Na) have been carried out at several C/n rates, where *n* is the number of hours needed for the insertion of two sodium per formula unit at the applied current intensity (1 C = 128 mA/g). The specific capacity has been calculated based on the mass of the active phase NFVPS in the positive electrodes. The cycling stability has been determined with a rate of C/2, while the rate performance has been studied at rates ranging from C/10 to 5C and then to 1C at 20 °C and 40 °C.

The chemicals PVDF, Na-electrolyte and N-methyl-2-pyrrolidone have been purchased from Sigma-Aldrich (St. Louis, MO, USA).

## 4. Conclusions

Adding carbon black (C) and reduced graphene oxide (rGO) to the NASICON-type multi-electron electrode, NFVPS, is crucial for its electrochemical performance. This complex phenomenon is a consequence of the oxygen-containing functional groups and porosity of the carbon additives. The carbon black contains predominately hydroxyl/epoxide functional groups, while rGO has a diversity of functional groups, namely hydroxyl/epoxide and carbonyl functional groups. Thus, both carbon black and rGO adhere well to the NFVPS particles, improving the thermal stability of NFVPS in the composites. Among carbon additives, the pore structure of NFVPS/rGO is more developed than that of NFVPS/C.

Sodium storage in carbon-modified NFVPS occurs through capacitive and Faradaic reactions. The Faradaic reaction is facilitated by the carbon black composite, while the capacitive reaction dominates in the rGO composite. NFVPS operates through two-electron reactions at 20 °C, while the increased temperatures favor the three-electron reaction. At 20 °C, the rGO composite outperforms the carbon composite in terms of cycling stability and rate capability. The discharge capacity remains at around 60 mAh/g at a 1C rate for 100 cycles. Increased temperature from 20 to 40 °C improves the performance of the rGO composite: the discharge capacity is around 95 mAh/g at a C/2 rate, and it maintains 95% cycling stability after 100 cycles. At 40 °C, the capacity of NFVPS/C at lower charging rate (i.e., between C/5 and 1C) is higher than that of the NFVPS/rGO composite, but at 5C the capacities of the two composites are similar. This shows that rate capability of NFVPS/rGO is better even at elevated temperatures.

The structural stability of NFVPS during cycling is one factor that contributes to the cycling stability of the composites. Additionally, the reactivity of the functional groups of the carbon additives toward electrolyte salt and solvent determines the composition of electrochemical surface layers. The hydroxyl/epoxide rich surface of the carbon black-composite is the most probably responsible for the deposition of fluorides, as well as facilitates the degradation of the propylene carbonate via the formation of alkoxyalkyl compounds. For the rGO-composite, the carbonyl groups, in addition to the hydroxyl/epoxide groups, cause the deposition of oxofluorides. Propylene carbonate degrades on NFVPS/rGO, forming alkylcarbonate and alkyloxyalkyl compounds.

This study demonstrates the multiple role of the carbon additives in the improving the sodium storage performance of the NASICON-type electrodes.

## Figures and Tables

**Table 1:** Ratio between the areas of the corresponding peaks from the deconvoluted spectra in the C1s region (Figure 3).

№	Electrodes	$\frac{C–O}{C–C}$	$\frac{C=O}{C–C}$
1	pristine NFVPS/rGO electrode	0.49	0.29
2	NFVPS/rGO electrode stopped at 1.5 V after 200 cycles: 100 cycles at 20 °C and next 100 cycles at 40 °C	0.61	0.25
3	pristine NFVPS/C electrode	0.33	0.08
4	NFVPS/C electrode stopped at 1.5 V after 200 cycles: 100 cycles at 20 °C and next 100 cycles at 40 °C	0.83	0.69

## Data Availability

Data available on request.

## Supplementary Materials

The following supporting information can be downloaded at: https://www.mdpi.com/article/10.3390/molecules30173547/s1, Figure S1: Crystal structure of NFVPS (a); Rietveld refinements plots of NFVPS (b), NFVPS/rGO (c) and NFVPS/C (d); Table S1: Lattice parameters of powders NFVPS, NFVPS/rGO and NFVPS/C and of electrodes cycled in Na half-cells between 1.5 and 4.5 V in NaPF_6_/PC electrolyte with C/2 rate; Figure S2: XPS spectra in the regions of O1s binding energies of pristine NFVPS/rGO electrode (a) and after 200 cycles in Na-half cell (100 cycles at 20 °C and subsequent 100 cycles at 40 °C with a rate C/2) (b); pristine NFVPS/C electrode (c) and after 200 cycles in Na-half cell (100 cycles at 20 °C and subsequent 100 cycles at 40 °C with a rate C/2) (d); Figure S3: Nitrogen adsorption/desorption isotherms (full/open symbols) with pore size distributions (insets) of NFVPS, NFVPS/rGO and NFVPS/C; Figure S4: CV curve of NFVPS/C and NFVPS/rGO between 1.5 and 5.0 V with a scanning rate of 10 mV/s at 20 °C in Na half-cell; Figure S5: Optical images of initial NFVPS/C and NFVPS/rGO electrodes and after cycling in Na half-cells at C/2 rate for 200 cycles (100 cycles at 20 °C and subsequent 100 cycles at 40 °C); Table S2: Element content determined from XPS spectra of the electrodes cycled between 1.5 and 4.5 V in Na half-cells with NaPF_6_/PC electrolyte.
