# Supplementary material for: Multiple Functions of Carbon Additives in NASICON-Type Electrodes for Stabilizing the Sodium Storage Performance
Source: Molecules. 2025 Aug 29;30(17):3547. doi: 10.3390/molecules30173547 (PMC12430351; doi:10.3390/molecules30173547)
Supplement: Supplementary file 1 [file molecules-30-03547-s001.zip › molecules-3822558-supplementary.pdf]

Multiple Functions of Carbon Additives in NASICON-type Electrodes for Stabilizing the Sodium Storage Performance

Trajche Tushev [1], Sonya Harizanova [1], Maria Shipochka [1], Radostina Stoyanova [1,2] and Violeta Koleva [1]*

[1]Institute of General and Inorganic Chemistry, Bulgarian Academy of Sciences, Acad. G. Bonchev Str., Bldg. 11, 1113 Sofia, Bulgaria;
tushev@svr.igic.bas.bg (T.T.); sonya@svr.igic.bas.bg (S.H.); shipochka@svr.igic.bas.bg (M.S.); radstoy@svr.igic.bas.bg (R.S.);
[2]National Centre of Excellence Mechatronics and Clean Technologies, Acad. G. Bonchev Str., Bldg. 29, 1113 Sofia, Bulgaria; radstoy@svr.igic.bas.bg (R.S.)
Correspondence: vkoleva@svr.igic.bas.bg (V.K.)

The powder materials $NaFeVPO_4(SO_4)_2$ and the composites $NaFeVPO_4(SO_4)_2$/rGO and $NaFeVPO_4(SO_4)_2$/C are denoted as NFVPS, NFVPS/rGO and NFVPS/C, respectively.

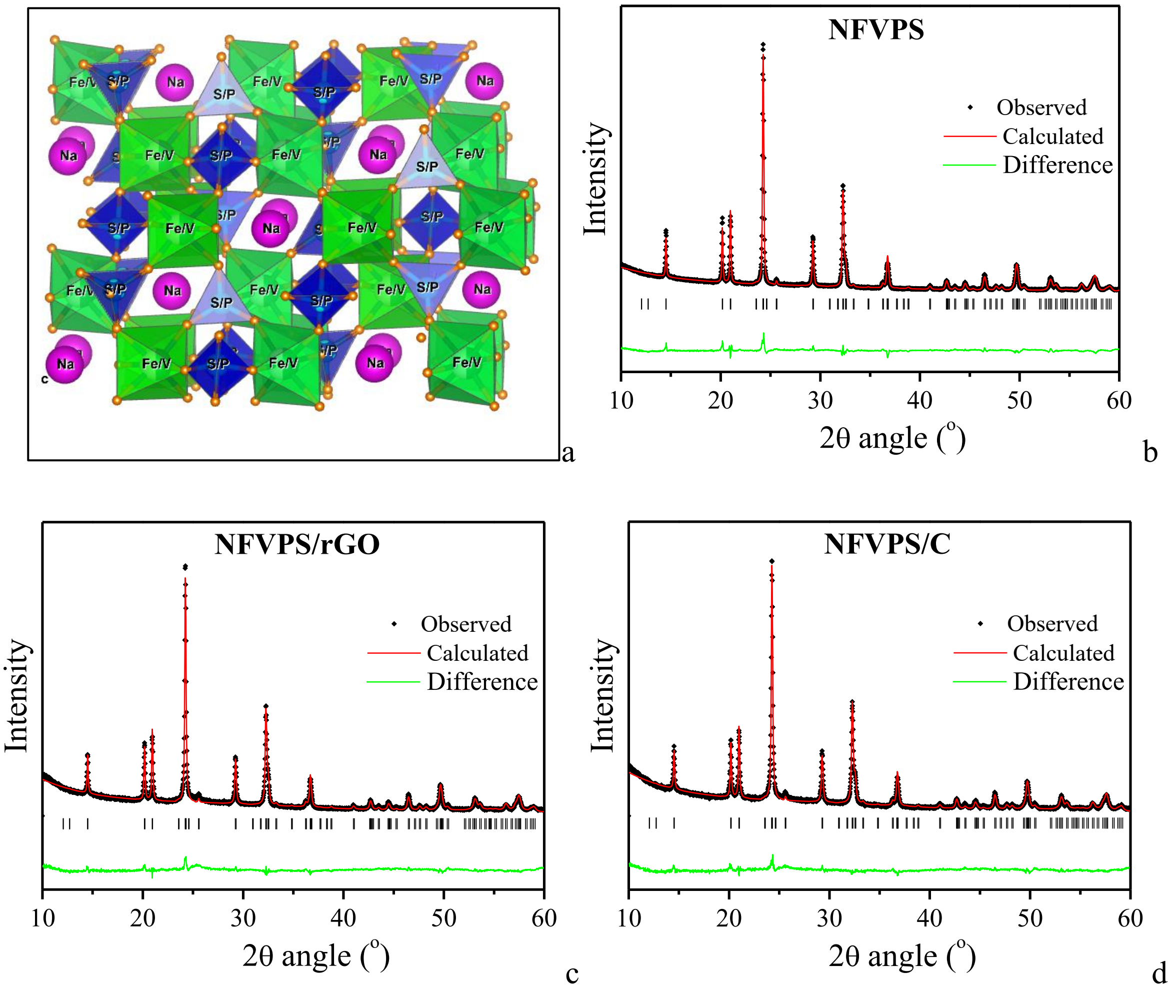

Figure S1. Crystal structure of NFVPS (a); Rietveld refinements plots of NFVPS (b), NFVPS/rGO(c) and NFVPS/C (d).

Table S1. Lattice parameters of powders NFVPS, NFVPS/rGO and NFVPS/C and of electrodes cycled in Na half-cells between 1.5 and 4.5 V in $NaPF_6$/PC electrolyte with C/2 rate

**Samples**	***a*** **(Å)**	***c*** **(Å)**	***V*** **(Å^3)**
NFVPS-powder	8.4691(1)	22.0162(1)	1367.58(1)
NFVPS/rGO - powder	8.4811(1)	21.9907(1)	1369.86(2)
NFVPS/C - powder	8.4634(1)	22.0158(1)	1365.72(2)
Pristine electrode NFVPS/rGO	8.4936(2)	21.9867(1)	1373.6(1)
NFVPS/rGO electrode after 200 cycles: 100 cycles at 20°C and subsequent 100 cycles at 40 °C	8.5088(2)	21.968(3)	1377.4(2)
Pristine electrode NFVPS/C	8.4664(1)	21.9936(2)	1365.3(1)
NFVPS/C electrode after 200 cycles: 100 cycles at 20°C and subsequent 100 cycles at 40 °C	8.4865(2)	21.9304(4)	1367.8(2)

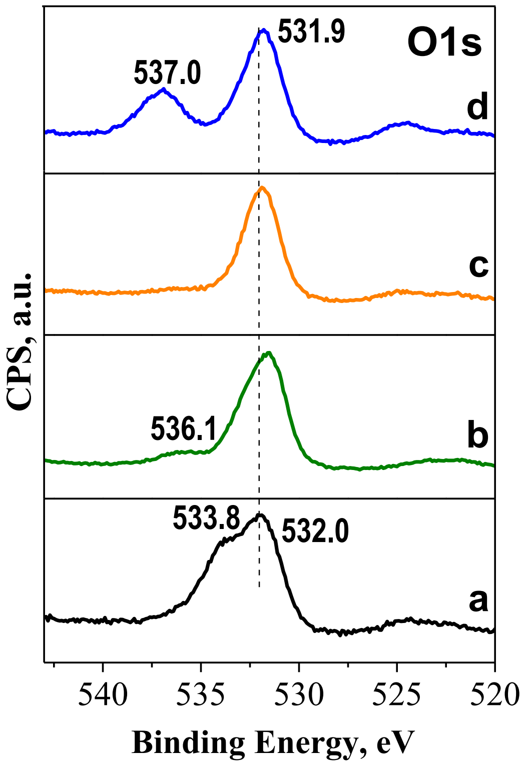

Figure S2. XPS spectra in the regions of O1s binding energies of pristine NFVPS/rGO electrode (a) and after 200 cycles in Na-half cell (100 cycles at 20 °C and subsequent 100 cycles at 40 °C with a rate C/2) (b); pristine NFVPS/C electrode (c) and after 200 cycles in Na-half cell (100 cycles at 20 °C and subsequent 100 cycles at 40 °C with a rate C/2) (d)

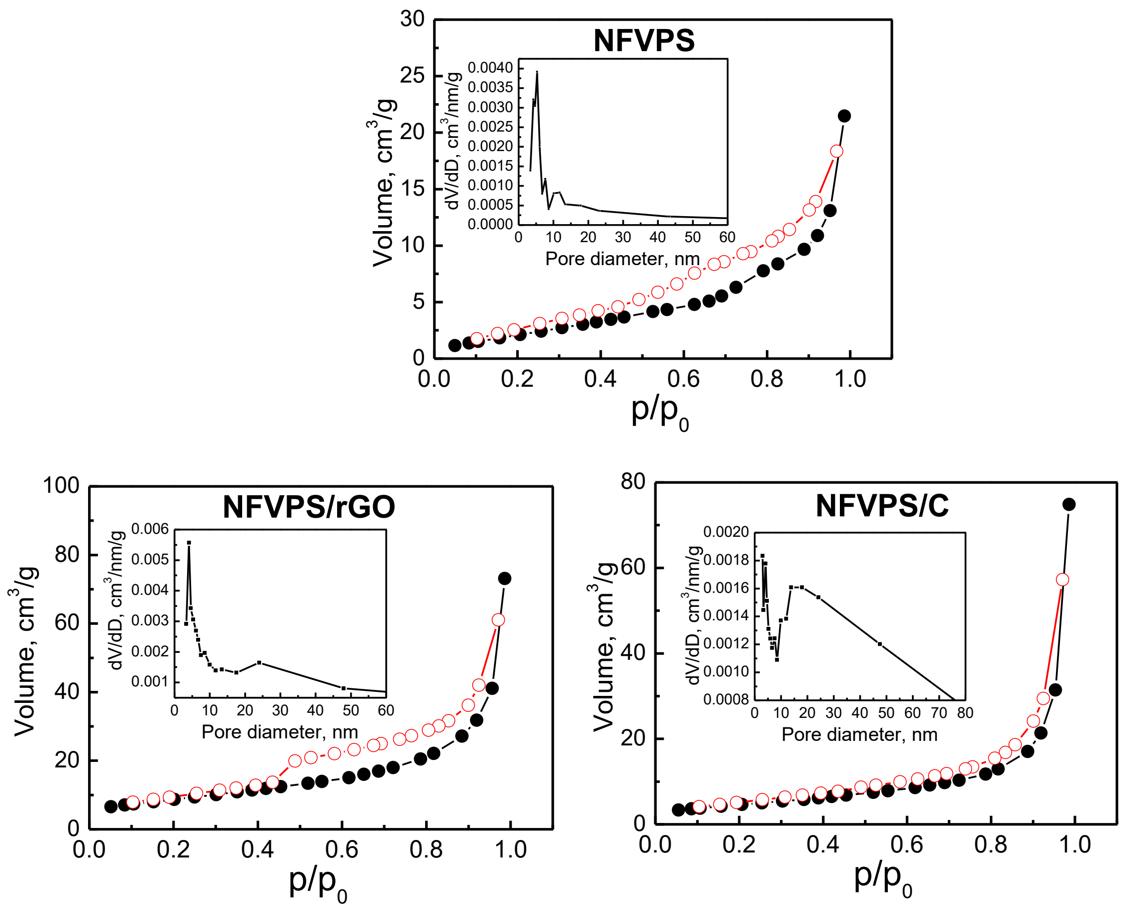

Figure S3. Nitrogen adsorption/desorption isotherms (full/open symbols) with pore size distributions (insets) of NFVPS, NFVPS/rGO and NFVPS/C

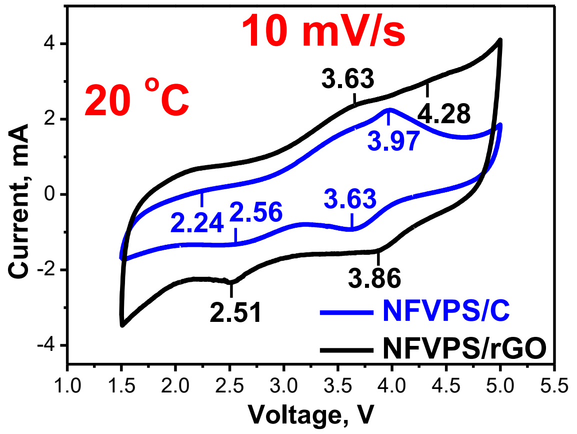

Figure S4. CV curve of NFVPS/C and NFVPS/rGO between 1.5 and 5.0 V with a scanning rate of 10 mV/s at 20 °C in Na half-cell.

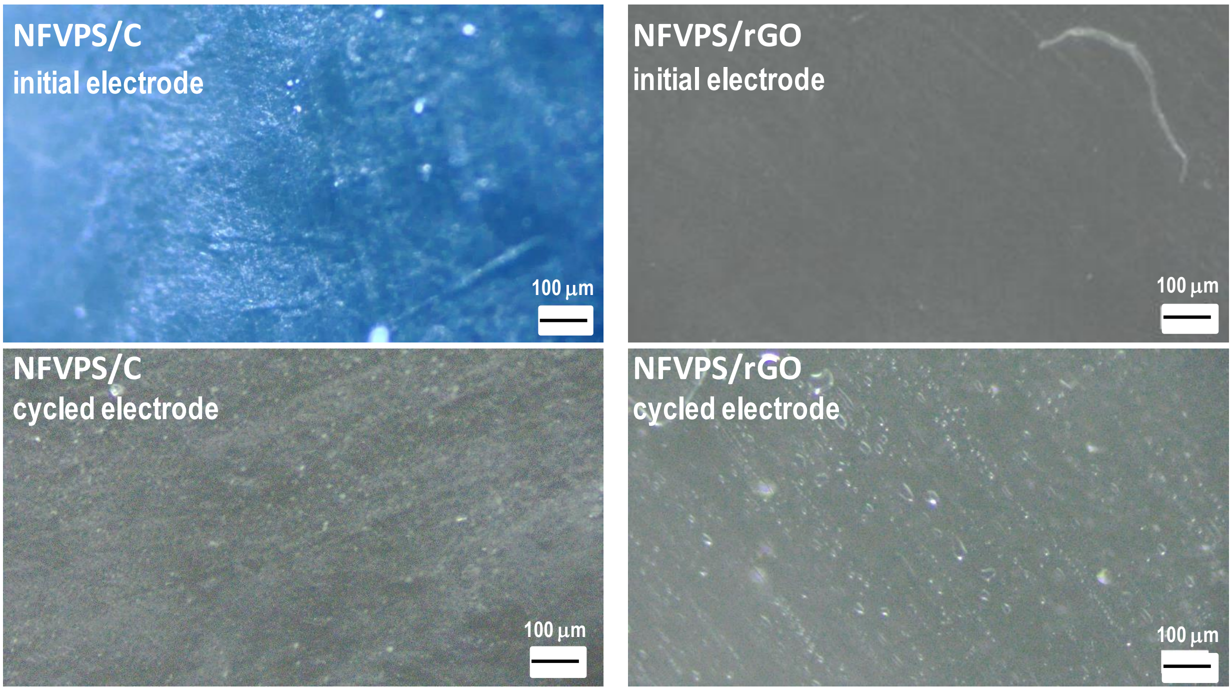

Figure S5. Optical images of initial NFVPS/C and NFVPS/rGO electrodes and after cycling in Na half-cells at C/2 rate for 200 cycles (100 cycles at 20 °C and subsequent 100 cycles at 40 °C).

Table S2. Element content determined from XPS spectra of the electrodes cycled between 1.5 and 4.5 V in Na half-cells with $NaPF_6$/PC electrolyte

№	Electrodes	Element content, at. %							
		C	O	V	Fe	P	S	F	Na
1	pristine NFVPS/rGO electrode	54.8	21.1	1.3	1.3	2.4	1.5	16.3	1.2
3	NFVPS/rGO electrode stopped at 1.5V after 200 cycles: 100 cycles at 20°C and next 100 cycles at 40 °C	20.5	44.9	0.6	1.8	8.3	3.2	11.6	9.1
3	pristine NFVPS/C electrode	58.5	16.0	1.2	1.4	3.1	1.7	17.1	1.0
4	NFVPS/C electrode stopped at 1.5V after 200 cycles: 100 cycles at 20°C and next 100 cycles at 40 °C	13.5	36.4	0.5	1.9	4.0	4.5	22.3	16.8
